# Functional Characterization of Triclosan-Resistant Enoyl-acyl-carrier Protein Reductase (FabV) in *Pseudomonas aeruginosa*

**DOI:** 10.3389/fmicb.2016.01903

**Published:** 2016-11-29

**Authors:** Yong-Heng Huang, Jin-Shui Lin, Jin-Cheng Ma, Hai-Hong Wang

**Affiliations:** ^1^Guangdong Provincial Key Laboratory of Protein Function and Regulation in Agricultural Organisms, College of Life Sciences, South China Agricultural UniversityGuangzhou, China; ^2^Shaanxi Engineering and Technological Research Center for Conversation and Utilization of Regional Biological Resources, College of Life Sciences, Yan’an UniversityYan’an, China

**Keywords:** *Pseudomonas aeruginosa*, enoyl-acyl-carrier protein reductase, triclosan, acylhomoserine lactones, swarming

## Abstract

*Pseudomonas aeruginosa* is extremely resistant to triclosan. Previous studies have shown that *P. aeruginosa* encodes a triclosan-resistant enoyl-acyl-carrier protein reductase (ENR), FabV, and that deletion of *fabV* causes *P. aeruginosa* to be extremely sensitive to triclosan. In this report, we complemented a *P. aeruginosa fabV* deletion strain with several triclosan-resistant ENR encoding genes, including *Vibrio cholerae fabV, Bacillus subtilis fabL* and *Enterococcus faecalis fabK*. All complemented strains restored triclosan resistance to the level of the wild-type strain, which confirmed that triclosan-resistant ENR allows *P. aeruginosa* to be extremely resistant to triclosan. Moreover, *fabV* exhibits pleiotropic effects. Deletion of *fabV* led *P. aeruginosa* to show attenuated swarming motility, decreased rhamnolipid, pyoverdine and acyl-homoserine lactones (AHLs) production. Complementation of the *fabV* mutant with any one ENR encoding gene could restore these features to some extent, in comparison with the wild-type strain. Furthermore, we found that addition of exogenous AHLs could restore the *fabV* mutant strain to swarm on semisolid plates and to produce more virulence factors than the *fabV* mutant strain. These findings indicate that deletion of *fabV* reduced the activity of ENR in *P. aeruginosa*, decreased fatty acid synthesis, and subsequently depressed the production of AHLs and other virulence factors, which finally may led to a reduction in the pathogenicity of *P. aeruginosa*. Therefore, *fabV* should be an ideal target for the control of *P. aeruginosa* infectivity.

## Introduction

*Pseudomonas aeruginosa* is an aerobic Gram-negative bacterium, which is widespread in the terrestrial environment (Driscoll et al., 2007; Lee and Zhang, 2015). As an important human pathogen, *P. aeruginosa* is responsible for a myriad of infections of the human body and is also a leading cause of mortality and morbidity in patients with cystic fibrosis (CF; Driscoll et al., 2007; Willcox, 2007; Kerr and Snelling, 2009). These infections are hard to eradicate because *P. aeruginosa* has developed strong resistance to most conventional antibiotics. The problem is further become complicated by the ability of the pathogen to form a biofilm matrix, which provides bacterial cells with a protected environment and allows them to withstand various stresses including antibiotics (Driscoll et al., 2007; Lee and Zhang, 2015). The breadth of difficult-to-treat *P. aeruginosa*-related infections makes the development of new anti-pseudomonas drugs a challenging priority. Fatty acid synthesis (FAS) is a vital metabolic pathway central to both mammals and bacteria (Campbell and Cronan, 2001; White et al., 2004; Zhang and Rock, 2008). Therefore, the divergence between mammalian and bacterial FAS pathway makes bacterial FAS an attractive target for the development of new antimicrobial agents (Campbell and Cronan, 2001; Heath et al., 2001; White et al., 2004).

**FIGURE 1:**
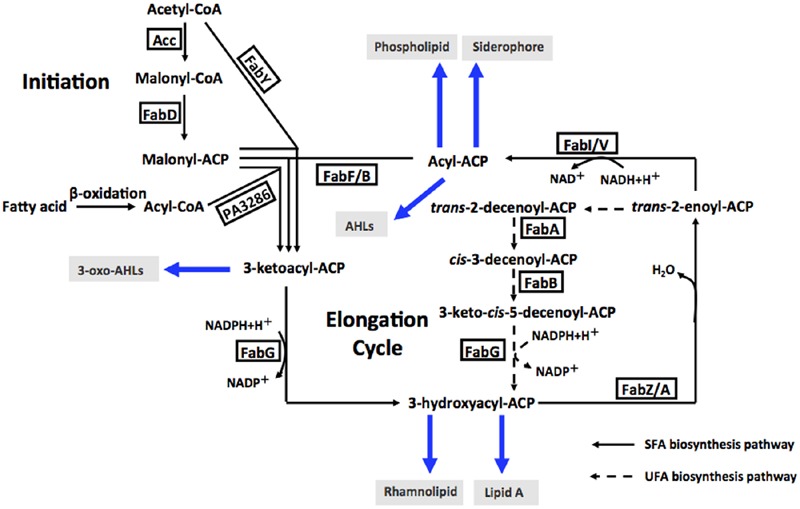
**Fatty acid biosynthesis in *P. aeruginosa*, and acyl-ACPs as acyl donors in cellular metabolism and AHL synthesis.** Abbreviations: Acc, acetyl-CoA carboxylase; FabD, malonyl-CoA:ACP transacylase; FabY, 3-ketoacyl ACP synthase III; PA3286, a new3-ketoacyl ACP synthase III; FabG, 3-ketoacyl-ACP reductase; FabZ, 3-hydroxyacyl-ACP dehydratase; FabA, 3-hydroxydecanoyl-ACP dehydratase/isomerase; FabF, 3-ketoacyl-ACP synthase II; FabB, 3-ketoacyl-ACP synthase I; FabI and FabV, enoyl-ACP reductase; AHLs, acylhomoserine lactones; SFA, saturated fatty acid; UFA, unsaturated fatty acid.

Enoyl-acyl-carrier protein reductase (ENR) is a vital enzyme in the bacterial fatty acid synthetic pathway, which catalyzes the last reduction of *trans*-2-acyl-ACP (an enoyl-ACP) to the fully saturated acyl-ACP species in the fatty acid elongation cycle (Massengo-Tiasse and Cronan, 2009) (**Figure 1**). Unlike most enzymes involved in type II fatty acid synthesis, ENRs display extensive sequence and structural diversity among bacteria. Based on the differing sensitivities of bacteria to triclosan, a biocide used in hand soaps and a large variety of other everyday products, four distinct ENR isozymes, FabI (Heath et al., 2001), FabL (Heath et al., 2000), FabK (Heath and Rock, 2000; Marrakchi et al., 2003) and FabV (Massengo-Tiasse and Cronan, 2008), have been identified. FabI has been shown to be the site of action of triclosan (Massengo-Tiasse and Cronan, 2009), while FabL, FabV and FabK are all triclosan-resistant ENRs. The fatty acid biosynthetic pathway of *P. aeruginosa* has been investigated extensively (Heath and Rock, 1995; Hoang and Schweizer, 1997; Hoang et al., 2002; Zhu et al., 2010; Yuan et al., 2012a,b) (**Figure 1**). In addition to FabI, *P. aeruginosa* also encodes a second ENR, FabV (Zhu et al., 2010).

*Pseudomonas aeruginosa* is extremely resistant to triclosan: the minimum inhibitory concentration (MIC) of triclosan for the wild-type strain is above 2000 μg/ml (Zhu et al., 2010). But deletion of *fabV* from the genome causes *P. aeruginosa* to become extremely sensitive to triclosan (>2,000-fold more sensitive than the wild-type strain). Therefore, it has been suggested that FabV is responsible for the inherent triclosan resistance of *P. aeruginosa* (Zhu et al., 2010). However, there is no more evidence to support this view.

In *P. aeruginosa*, FabV also exhibits pleiotropic effects (Bai et al., 2007, 2011; Mou et al., 2011). The *fabV* (formerly named *pfm*) gene was first identified to be required for swimming motility, and it was suggested that FabV was involved in energy metabolism, which is critical for the rotation of the flagellum in *P. aeruginosa* (Bai et al., 2007). Further studies showed that *fabV* was involved in bacterial protein secretion and bacterial adherence, and mutation of *fabV* caused *P. aeruginosa* to produce significantly fewer QS signal molecules and reduced the pathogenicity and virulence in a murine model of acute lung infection. Thus, *fabV* was suggested to be an important target for the control of *P. aeruginosa* infectivity (Bai et al., 2011; Mou et al., 2011). However, the mechanism of involvement of FabV in the pathogenicity of *P. aeruginosa* is still not well studied.

In *P. aeruginosa*, FAS not only supplies the precursors for phospholipid and lipopolysaccharide synthesis, but also shunt the intermediates to produce siderophores, fatty acid-dependent quorum-sensing signals (PQS and homoserine lactones) and rhamnolipids (Hoang et al., 2002). Thus, we hypothesized that deletion of *fabV* would reduce the activity of ENR in *P. aeruginosa*, and subsequently reduce FAS, and then depress the production of QS signals and other virulence factors, and finally would lead to reduced pathogenicity in *P. aeruginosa*.

In this study, we provided evidence to demonstrate that it is FabV that confers triclosan resistance on *P. aeruginosa* by complementation of a *fabV* deletion mutant with *Vibrio cholerae fabV, Enterococcus faecalis fabK* and *Bacillus subtilis fabL*. We also confirmed our hypothesis by assaying the fatty acid synthetic rate of the *fabV* mutant, by measurement of the production of several end products that are related to FAS in the *fabV* mutant, and by addition of exogenous AHLs to restore the phenotype of the *fabV* mutant.

## Materials and Methods

The supply sources were as follows: fatty acids (FAs), triclosan, rhamnose, N-butyryl-homoserine lactone (C_4_-HSL), N-3-oxo-dodecanoyl-homoserine lactone (3-oxo-C_12_-HSL), NADH and antibiotics were from Sigma-Aldrich; Takara Biotechnology Co. provided molecular biology reagents; and Bio-Rad provided the Quick Start Bradford dye reagent. All other reagents were of the highest available quality.

### Bacterial Strains, Plasmids, and Growth Media

The strains and plasmids used in this study are listed in Supplementary Table S1. The *E. coli* K-12 strain DH5α was used in this study for gene cloning. The *P. aeruginosa* strains used in this study were the wild-type strain PAO1, *fabV* deletion strain PAO170 and *fabI* deletion strain PAO272. Luria-Bertani (LB) medium was used as the enriched medium for *E. coli* and *P. aeruginosa* growth. Antibiotics were used at the following concentrations (in micrograms per milliliter): sodium ampicillin, 100 (for *E. coli*); kanamycin sulfate, 30 (for *E. coli*) or 100 (for *P. aeruginosa*); gentamicin, 10 (for *E. coli*) or 100 (for *P. aeruginosa*); and triclosan, 3 (for *P. aeruginosa*). Isopropyl-β-D-thiogalactoside (IPTG) was used at a final concentration of 1 mmol/L.

### Recombinant DNA Techniques and Construction of Plasmids

*Pseudomonas aeruginosa fabI* (*PafabI*) and *fabV* (*PafabV*), *V. cholerae fabV* (*VcfabV*), *E. faecalis fabK* (*EnfabK*) and *B. subtilis fabL* (*BsfabL*) were amplified from genomic DNA obtained from wild-type strains of *P. aeruginosa, V. cholerae, E. faecalis* and *B. subtilis*, respectively. The primers are listed in Supplementary Table S2. The PCR was performed with *Pfu* DNA polymerase and the products were inserted into T-vector pMD19, to give plasmids pYH1 (*PafabI*), pYH2 (*PafabV*), pYH3 (*VcfabV*), pYH4 (*EnfabK*) and pYH5 (*BsfabL*), respectively. All the genes in the T-vectors were confirmed by sequencing, performed by Shanghai Sangon, Inc. The *fab* genes in T-vector were digested with NdeI and HindIII, and the appropriate fragments were isolated and ligated into pSRK-Km (Khan et al., 2008) to create plasmids pSRK-PI, pSRK-PV, pSRK-VV, pSRK-EK and pSRK-BL, respectively. All these plasmids were introduced into the *P. aeruginosa* PAO170 strain via electroporation to give strains: PI170 (PAO170/pSRK-PI), PV170 (PAO170/ pSRK-PV), VV170 (PAO170/pSRK-VV), EK170 (PAO170/pSRK-EK) and BL170 (PAO170/ pSRK-BL), respectively.

### Analysis of Fatty Acid Composition of Bacteria

The cellular lipid assay was adapted from that of Stead (1989). Briefly, cultures of the *P. aeruginosa* strains were grown at 37°C in LB medium overnight. Cells were then harvested from 10 ml aliquots of the cultures and washed with fresh LB medium at room temperature. Cellular lipids were saponified by the addition of 1 ml NaOH in methanol solution (NaOH 45 g; methanol 150 ml; water 150 ml). The samples were placed in a boiling water bath for 30 min. The tubes were vortexed before and once during boiling. FAs were methylated by the addition of 2 ml of 6 mol/L HCl in methanol (325 ml 11.6 mol/L HCl, plus 275 ml methanol). The samples were heated at 80°C for 10 min and immediately cooled to below 20°C. The fatty acid methyl esters were extracted three times with 1.25 ml petroleum ether. The samples were dried under a stream of nitrogen in a fume hood. The esters were analyzed by gas chromatography-mass spectrometry (GC-MS) as described previously (Feng et al., 2015; Mao et al., 2016). The data are presented as percentages of the total FAs and represent the mean ± standard error for three independent determinations.

### Enoyl-ACP Reductase Assays

Cell-free extracts of *P. aeruginosa* were prepared from early stationary phase growing cultures (optical density at 600 nm of 0.8 to 1.0). Cells grown in LB medium at 37°C were harvested by centrifugation and were then suspended in lysis buffer (0.1 mol/L sodium phosphate, pH 7.5, 5 mmol/L β-mercaptoethanol, 1 mmol/L EDTA). The cell lysates were prepared by passing the cell suspensions three times through a French pressure cell. Cell debris was removed by ultracentrifugation for 1 h at 260,000 × *g*, and the supernatants were dialyzed against lysis buffer for 24 h and saved as cell extracts. The ENR activity of cell-free extracts was determined by using *trans*-2-decenoyl-ACP as the substrate, which was done by monitoring spectrophotometrically the decrease in the absorbance at 340 nm using an NADH extinction coefficient of 6,220 mol/L^-1^. The *trans*-2-decenoyl-ACP was prepared by using a previously published procedure (Zhu et al., 2010). The reaction mixtures for activity assays contained 150 μmol/L NADH, 10 ng of the cell-free extracts, 100 μmol/L *trans*-2-decenoyl-ACP, and 0.1 mol/L LiCl in 0.1 mol/L sodium phosphate buffer (pH 7.0).

### Motility Assays

The swarming, swimming, and twitching motilities of *P. aeruginosa* were investigated using the following media: (1) swim plate [1% tryptone, 0.5% NaCl, 0.3% agar (Difco Bacto Agar)], (2) swarm plate (0.45% tryptone, 0.13% yeast extract, 0.22% NaCl, 0.5% glucose, 0.5% agar), and (3) twitch plate (1% tryptone, 0.5% yeast extract, 0.5% NaCl, 1% agar). The agar media were air-dried for 5–10 min before use. For the swimming and swarming assays, plates were point-inoculated with bacteria from an overnight culture using a sterile toothpick on the surface, and the plates were incubated at 30°C for 24–48 h. For the twitching motility assay, the cells were stabbed into the bottom of twitching plates using a toothpick and incubated at 37°C for 24 h. The motility was assessed by examining the circle around the inoculation site formed by the growing bacteria.

### Rhamnolipid Assay

For the rhamnolipid assay, *P. aeruginosa* strains were cultured to stationary phase in PPGAS (120 mmol/L Tris-HCl, pH 7.2, 20 mmol/L NH_4_Cl, 20 mmol/L KCl, 1.6 mmol/L MgSO_4_, 1% tryptone and 0.5% glucose) at 30°C. The supernatants were first adjusted to pH 2.0 with 1 mol/L HCl, and 1 ml of these supernatant samples was extracted twice with an equal volume of diethyl ether, followed by vacuum drying. The concentration of rhamnolipid was determined by measuring the concentration of rhamnose with the sulfuric acid-anthrone reagent (0.2% anthrone, 85% sulfuric acid) method, using rhamnose sugar as the standard, at 620 nm.

### Siderophore Secretion Assay

Chrome azurol S (CAS)-LB agar plates were used for the siderophore secretion assay: 10 ml sterilized 10X CAS solution (1 mmol/L chrome azurol S, 2 mmol/L cetyltrimethylammonium bromide, 500 μmol/L FeCl_3_⋅6H_2_O) was mixed with 100 ml LB agar to prepare CAS-LB agar plates. The plates were dried for 1 h at room temperature prior to inoculation, following which 10 μl of overnight *P. aeruginosa* culture was drop inoculated onto a large CAS-LB agar plate (ø 200 mm). Transparent circles were observed after 48 h of incubation at 37°C.

### Extraction and Assay of Quorum-Sensing Signal Molecules

Cultures were grown in LB medium for 24 h at 37°C with shaking (250 rpm) to stationary phase. Supernatants were harvested by centrifugation (12,000 × *g*, 5 min at room temperature), and 5 ml of supernatant for each sample was extracted using an equal volume of ethyl acetate. The organic phase was concentrated to dryness. For the N-butyryl-L-homoserine lactone (C_4_-HSL) assay, 10 μl of each sample resolved in 50 μl ethyl acetate was absorbed by a ø 0.5 cm filter paper and then tiled on an LB agar plate, which was newly covered with *Chromobacterium violaceum* CV026. Purple circles were observed after overnight incubation at 30°C. To detect N-3-oxo-dodecanoyl-L-homoserine lactone (3-oxo-C_12_-HSL) production, the dry extracts were resolved in 50 μl acetonitrile and 10 μl of each sample was analyzed with HPLC according to the previously published procedure (Ortori et al., 2007). In addition, 10 mmol/L pure 3-oxo-C_12_-HSL (Sigema) acetonitrile solution was used as a reference.

### Pyocyanin Quantitation Assay

The pyocyanin assay is based on the absorbance of pyocyanin at 520 nm in acidic solution. A 5-ml sample of *P. aeruginosa* culture grown in LB was extracted with 3 ml of chloroform and then re-extracted into 1 ml of 0.2 mmol/L HCl to give a pink to deep red solution. The absorbance of this solution was measured at 520 nm. Concentrations, expressed as micrograms of pyocyanin produced per milliliter of culture supernatant, were determined by multiplying the optical density at 520 nm by 17.072.

### LasA Protease Activity Assay

LasA protease activity was determined by measuring the ability of *P. aeruginosa* culture supernatants to lyse boiled *Staphylococcus aureus* cells. A 30 ml overnight culture of *S. aureus* was placed in a boiling water bath for 10 min and then centrifuged for 10 min at 10,000 × *g*. The resulting pellet was resuspended in 10 mmol/L Na_2_HPO_4_ (pH 7.5) and adjusted to an OD_600_ of 0.9. A 100 μL aliquot of *P. aeruginosa* supernatant was then added to 900 μL *S. aureus* suspension, and the OD_600_ was determined after 5, 10, 15, 20, 25, 30, 35, 40, 45, 60, 75, 90, and 105 min.

### Statistical Analyses

Analysis of variance for experimental datasets was performed using JMP soft ware, version 5.0 (SAS Institute Inc., Cary, NC, USA). Significant effects of treatment were determined by the *F* value. When a significant *F* test was obtained, separation of means was accomplished by Fisher’s protected LSD (least significant difference) at *P* ≤ 0.05.

## Results

### FabV is Responsible for to the Resistance of *P. aeruginosa* to Triclosan

**FIGURE 2:**
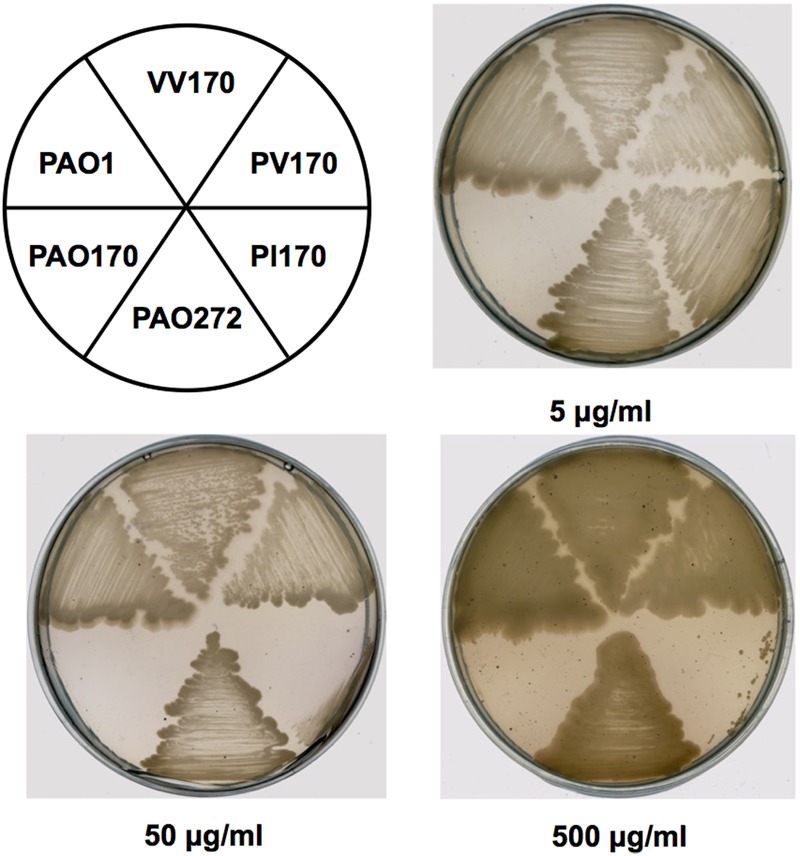
**Growth inhibition of *P. aeruginosa* strains by triclosan.** The concentration of triclosan is shown below each plate. PAO1 indicates *P. aeruginosa* wild-type strain PAO1; PAO170 indicates *P. aeruginosa fabV* deletion strain; PAO272 indicates *P. aeruginosa fabI* deletion strain; PI170 indicates strain PAO170 carrying plasmid pSRK-PI; PV170 indicates strain PAO170 carrying plasmid pSRK-PV; VV170 indicates strain PAO170 carrying plasmid pSRK-VV.

In order to confirm the role of FabV in *P. aeruginosa* resistance to triclosan, we tested the sensitivity to triclosan of PAO170 (Δ*fabV*) carried pSRK-PI (carries *P. aeruginosa fabI*), pSRK-PV (carries *P. aeruginosa fabV*) and pSRK-VV (carries *V. cholerae fabV*), respectively. As expected, the derivative of strain PAO170 carrying the vector plasmid (pSRK-Km) was very sensitive to triclosan (the triclosan MIC was about 1 μg/ml; **Figure 2** and **Table 1**). In contrast, expression of PaFabI, PaFabV, and VcFabV proteins from pSRK-Km-derived plasmids increased triclosan resistance. However, the MIC of strain PAO170 (Δ*fabV*) carrying plasmid pSRK-PI (5 μg/ml) was much lower than that of strain PAO170 (Δ*fabV*) carrying plasmid pSRK-PV or pSRK-VV (above 2,000 μg/ml), which was the same as that of the wild-type strain PAO1 (**Figure 2** and **Table 1**). These findings suggest that the sensitivity of mutant strain PAO170 (Δ*fabV*) to triclosan was due to its lack of triclosan-resistant ENR. To investigate this point further, we also introduced pSRK-EK (carries *E. faecalis fabK*) and pSRK-BL into *P. aeruginosa fabV* mutant strain PAO170, respectively. The test of sensitivity to triclosan showed that strain PAO170 (Δ*fabV*) carrying both plasmids was highly resistant to triclosan; the triclosan MIC was above 2,000 μg/ml (Supplementary Figure S1 and **Table 1**). These data confirmed that FabV, a triclosan-resistant ENR, leads to resistance of *P. aeruginosa* to triclosan.

**Table 1:** Triclosan resistance of *P. aeruginosa* strains.

Strains	Clone	Triclosan MIC (μg/ml)
PAO1	PAO1	>2000
PAO170 (*fabV*::Gm)	PAO170	1
PAO272 (*fabI*::Gm)	PAO272	>2000
PI170	PAO170/pSRK-PI	5
PV170	PAO170/pSRK-PV	>2000
VV170	PAO170/pSRK-VV	>2000
BL170	PAO170/pSRK-BL	>2000
EK170	PAO170/pSRK-EK	>2000

### Deletion of *fabV* Attenuated *P. aeruginosa* Swarming Motility

**FIGURE 3:**
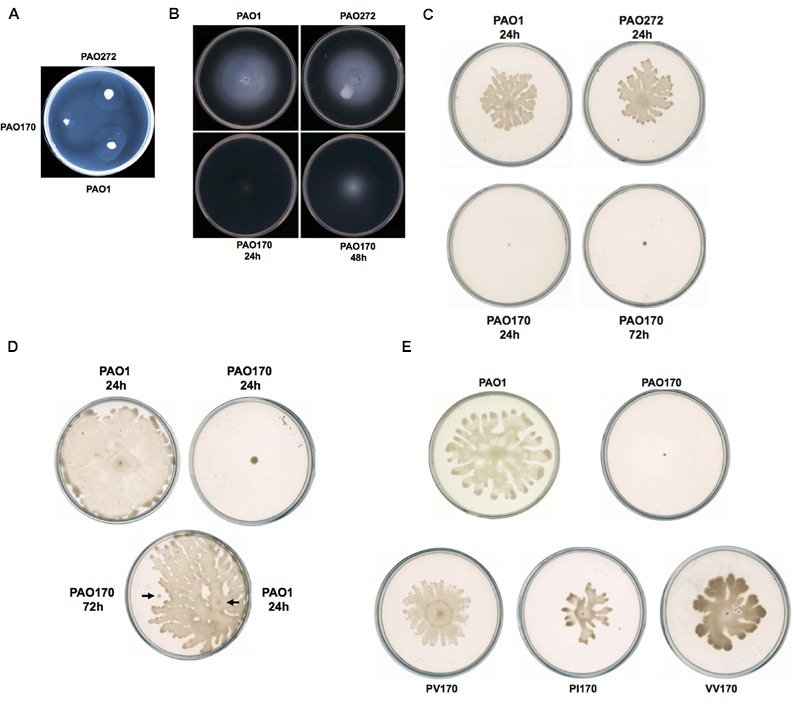
**Twitching, swimming, and swarming motility of *P. aeruginosa* strains. (A)** Twitching motility patterns of *P. aeruginosa* strains after incubation at 37°C for 24 h. **(B)** Swimming motility patterns of *P. aeruginosa* strains after incubation at 30°C for 24–48 h. **(C)** Swarming motility patterns of *P. aeruginosa* strains after incubation at 30°C for 24–72 h. **(D)** Swarming motility patterns of *P. aeruginosa* PAO170 grown on a swarming plate containing 0.4% agar. **(E)** Swarming motility patterns of *P. aeruginosa* PAO170 carrying ENR encoding genes.

Motility is strongly associated with the pathogenesis of *P. aeruginosa*. The bacterium exhibits movement on surfaces by utilizing three types of motility: swimming, swarming, and twitching (Inoue et al., 2008; Overhage et al., 2008). **Figure 3** shows the colony spreading patterns of these types of motility in *P. aeruginosa* mutant strains. The twitching pattern of mutant strain PAO272 (Δ*fabI*) was almost the same as that of wild-type strain PAO1, whereas strain PAO170 (Δ*fabV*) formed a much smaller twitching pattern than wild-type strain PAO1 (**Figure 3A**), which suggests that deletion of the *fabI* gene had no effect on twitching motility of *P. aeruginosa*, but deletion of *fabV* inhibited *P. aeruginosa* twitching motility.

Mutant strain PAO170 (Δ*fabV*) did not form a swimming pattern after 24 h of incubation on swimming assay plates at 30°C. However, when the incubation time was extended to 48 h, it was able to swim and form a typical swimming pattern, although the average swimming diameter of strain PAO170 (Δ*fabV*) was much smaller than that of the wild-type strain and the *fabI* mutant strain PAO272 (Δ*fabI*; **Figure 3B**). This result was not consistent with a previous study, in which Bai et al. (2007) reported that mini-Mu insertion in *fabV* caused the PA68 strain of *P. aeruginosa* to lose swimming motility. However, it is important to note that, when Bai et al. (2007) carried out the swimming assay for the *fabV* strain, they incubated the mutant strain for only 16 h on a swimming assay plate. Thus, we speculate that mutation of *fabV* did not inhibit *P. aeruginosa* swimming motility directly, but led to slow growth and therefore caused it to form the swimming pattern late. We tested the growth of strain PAO170 (Δ*fabV*) in swimming medium and confirmed that it grew more slowly than wild-type strain PAO1 (data not shown). Meanwhile, deletion of *fabI* had no effect on swimming motility of *P. aeruginosa* because, like wild-type strain PAO1, the mutant strain PAO272 (Δ*fabI*) was able to form a normal swimming pattern on the swimming assay plate after 24 h of incubation (**Figure 3B**).

The swarming motility of *P. aeruginosa* strains was also tested on semisolid plates (containing 0.5% agar) at 30°C (**Figure 3C**). Although both the *fabI* mutant strain PAO272 (Δ*fabI*) and the wild-type strain swarmed normally on semisolid plates after 24 h of incubation, the *fabV* mutant strain PAO170 failed to form a swarming pattern, even when the incubation time was extended to 48 h (**Figure 3C**). In swarming medium, the *fabV* mutant strain grew more slowly than wild-type strain PAO1 (data not shown). Therefore, to test whether the deficiency of swarming motility was due to the weak growth of the *fabV* mutant strain, we repeated the swarming assay on semisolid plates containing 0.4% agar. As expected, after 24 h of incubation the wild-type strain PAO1 formed obvious swarms on semisolid plates containing 0.4% agar, while the *fabV* mutant strain failed to form a swarming pattern (**Figure 3D**). Subsequently, using a toothpick, we inoculated wild-type strain PAO1 on a semisolid plate on which the *fabV* mutant strain had been cultured for 48 h. After a further 24 h of incubation, the PAO1 strain formed a swarming pattern, but the *fabV* strain still did not swarm (**Figure 3D**). These data indicate that it was disruption of *fabV* that caused *P. aeruginosa* to lose swarming motility. To test this hypothesis, we checked the swarming motility of complementary strain PAO170/pSRK-PV on a semisolid plate (containing 0.5% agar). Like wild-type strain PAO1, strain PAO170/pSRK-PV was able to form a typical swarming pattern after 24 h of incubation on the semisolid plate (**Figure 3E**). Moreover, it was interesting that not only *P. aeruginosa fabV* but also *P. aeruginosa fabI* could restore normal swarming motility to the *fabV* mutant (**Figure 3E**). We also tested whether *V. cholerae fabV, E. faecalis fabK* and *B. subtilis fabL* complemented the swarming motility of the *fabV* mutant. The data showed that all these ENR encoding genes were able to restore swarming motility to the *fabV* mutant (**Figure 3E** and Supplementary Figure S2). These results suggest that ENR is required for swarming motility in *P. aeruginosa*.

### The Activity of ENR is Essential to *P. aeruginosa* Growth

**FIGURE 4:**
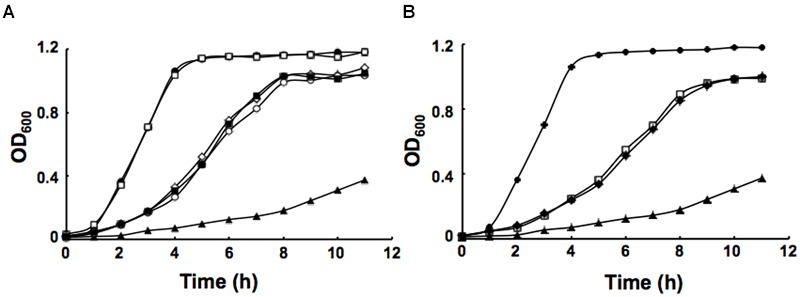
**Growth of *P. aeruginosa* strains in LB medium. (A)** Growth of the strains PAO1 (filled circle), PAO272 (empty square), PAO170 (filled triangle), PI170 (empty circle), PV170 (filled square) and VV170 (empty diamond). **(B)** Growth of the strains PAO1 (filled circle), EK170 (empty square), BL170 (filled diamond) and PAO170 (filled triangle). All cultures were grown at 37°C.

It has been reported that in defined minimal medium the *fabV* mutant strain PAO170 was viable, but its generation time (about 118 min) was longer than that of the wild-type strain PAO1 (about 40 min; Zhu et al., 2010). To determine whether nutrient conditions affect the growth of strain PAO170, its growth in LB medium was examined. The growth of stain PAO170 in LB medium was significantly slower than that of wild-type strain PAO1, confirming that nutrient conditions did not improve the growth of strain PAO170 (**Figure 4A**). Complementation of strain PAO170 with plasmid pSRK-PV increased the growth of PAO170 to a certain extent, but not to the level of the wild-type strain (**Figure 4A**). Plasmid pSRK-PI or pSRK-VV also allowed strain PAO170 to grow to the same level as pSRK-PV (**Figure 4A**). These results indicated that deletion of *PafabV* from the genome caused *P. aeruginosa* to grow slowly and that ENR encoding genes could restore growth of the *fabV* mutant to a certain extent. We also investigated the growth of PAO170 carrying *E. faecalis fabK* or *B. subtilis fabL* encoding plasmids and obtained similar results (**Figure 4B**). To investigate further possible reasons for the weak growth of strain PAO170, the fatty acid compositions of strain PAO170 were determined by GC-MS. Although the mutant strain PAO170 contained the same fatty acid species as the wild-type strain, PAO170 produced more 3-hydroxy fatty acids (3-HFAs) and fewer FAs (**Table 2**). The ratio of FAs to 3-HFAs in strain PAO170 was 2.58 and was lower than that of the wild-type strain (3.87), which showed significant difference (*P* < 0.05) between mutant strain PA170 and wild-type strain PAO1. Complementation of strain PAO170 with plasmid pSRK-PI or pSRK-VV restored the ability of strain PAO170 to produce the wild-type strain’s level of 3-HFAs and FAs. The ratio of FAs to 3-HFAs in strain PAO170 carrying pSRK-PI or pSRK-VV was 3.70 or 3.61, respectively. Moreover, strain PAO170 carrying pSRK-PV produced more FAs and fewer 3-HFAs than the wild-type strain. The ratio of FAs to 3-HFAs in strain PAO170 carrying pSRK-PV was 4.57 (**Table 2**), and there was highly significant difference between strain PV170 and PAO170 (*P* < 0.01). It is noteworthy that mutant strain PAO170 carrying pSRK-PV, pSRK-PI or pSRK-VV grew to the same level. This indicated that a change in fatty acid composition caused by deletion of *fabV* was not the main reason for weak growth in *P. aeruginosa fabV* mutant. Another possibility was that deletion of *fabV* led to decreased activity of ENR, which was a contributing factor in the lower growth rate of PAO170. We measured the activity of ENR in cell-free extract of PAO170 by using *trans*-2-decenoyl-ACP as a substrate and monitoring the decrease in NADH absorbance at 340 nm. The ENR activity (in μmol/min per mg extract protein) in cell-free extract of PAO170 was 0.175 ± 0.006, and was lower than that in the cell-free extract of wild-type strain PAO1 (0.478 ± 0.032). The ENR activity in strain PAO170 carrying plasmids pSRK-PI, pSRK-PV and pSRK-VV was 0.290 ± 0.001, 0.292 ± 0.004 and 0.300 ± 0.006, respectively. Although the ENR activity in strain PAO170 carrying plasmids with genes encoding foreign ENR was increased, it was still lower than that in wild-type strain PAO1. We also detected the ENR activity in cell-free extracts of PAO170 carrying pSRK-EK or pSRK-BL encoding plasmids. The ENR activity in both strains was increased, but did not reach the level of that in the wild-type strain either (data not shown). Therefore, we suggest that the level of activity of ENR, rather than the *PafabV* gene or protein product, is essential to the growth of *P. aeruginosa*.

**Table 2:** Fatty acid composition of total lipid extracts from the *P. aeruginosa*^α^.

Fatty acid	PAO1	PAO272	PAO170	PV170	PI170	VV170
n-3-OH-C_10:0_	7.96 ± 0.40	7.21 ± 0.11	10.62 ± 0.12	6.86 ± 1.32	7.65 ± 0.13	7.13 ± 0.10
n-C12:0	1.75 ± 0.31	1.09 ± 0.23	1.42 ± 0.36	2.75 ± 0.64	3.08 ± 0.17	2.69 ± 0.19
n-3-OH-C_12:0_	12.59 ± 0.73	13.79 ± 0.78	17.23 ± 1.37	11.12 ± 0.55	13.64 ± 0.22	14.53 ± 1.99
n-C14:0	0.85 ± 0.04	0.69 ± 0.06	0.75 ± 0.05	1.23 ± 0.01	1.20 ± 0.05	1.08 ± 0.06
n-C16:1 *cis*-9	10.74 ± 2.07	11.03 ± 0.14	9.36 ± 0.44	12.27 ± 0.26	10.11 ± 0.74	8.38 ± 0.24
n-C16:0	31.51 ± 2.17	30.62 ± 0.59	27.92 ± 1.45	35.54 ± 0.52	35.12 ± 0.58	33.24 ± 2.62
n-C18:1 *cis*-11	32.5 ± 0.47	32.97 ± 0.29	30.27 ± 0.77	28.21 ± 1.57	27.01 ± 0.63	30.92 ± 2.76
n-C18:0	2.11 ± 0.20	2.59 ± 0.43	2.38 ± 0.99	2.04 ± 0.08	2.19 ± 0.43	2.02 ± 0.06
FA/HFA	3.87^∗^	3.76^∗^	2.58	4.57^∗∗^	3.70^∗^	3.61^∗^

*^α^Cells were grown on RB plates overnight at 42°C. The total lipids were extracted and transesterified to obtain fatty acid methyl esters, and products were identified by GC-MS. The values are the means ± standard deviations of three independent experiments and percentages of total fatty acids. Pair-wise comparisons were made between mutant strain PAO170 and wild-type strain PAO1 or complemented strains, such as, PV170, PI170 and VV170, by Student’s *t*-test. ^∗∗^, Highly significant difference, *P* < 0.01. ^∗^, Significant difference, *P* < 0.05.*

### Deletion of *fabV* Muted the Production of Several Exo-products

**FIGURE 5:**
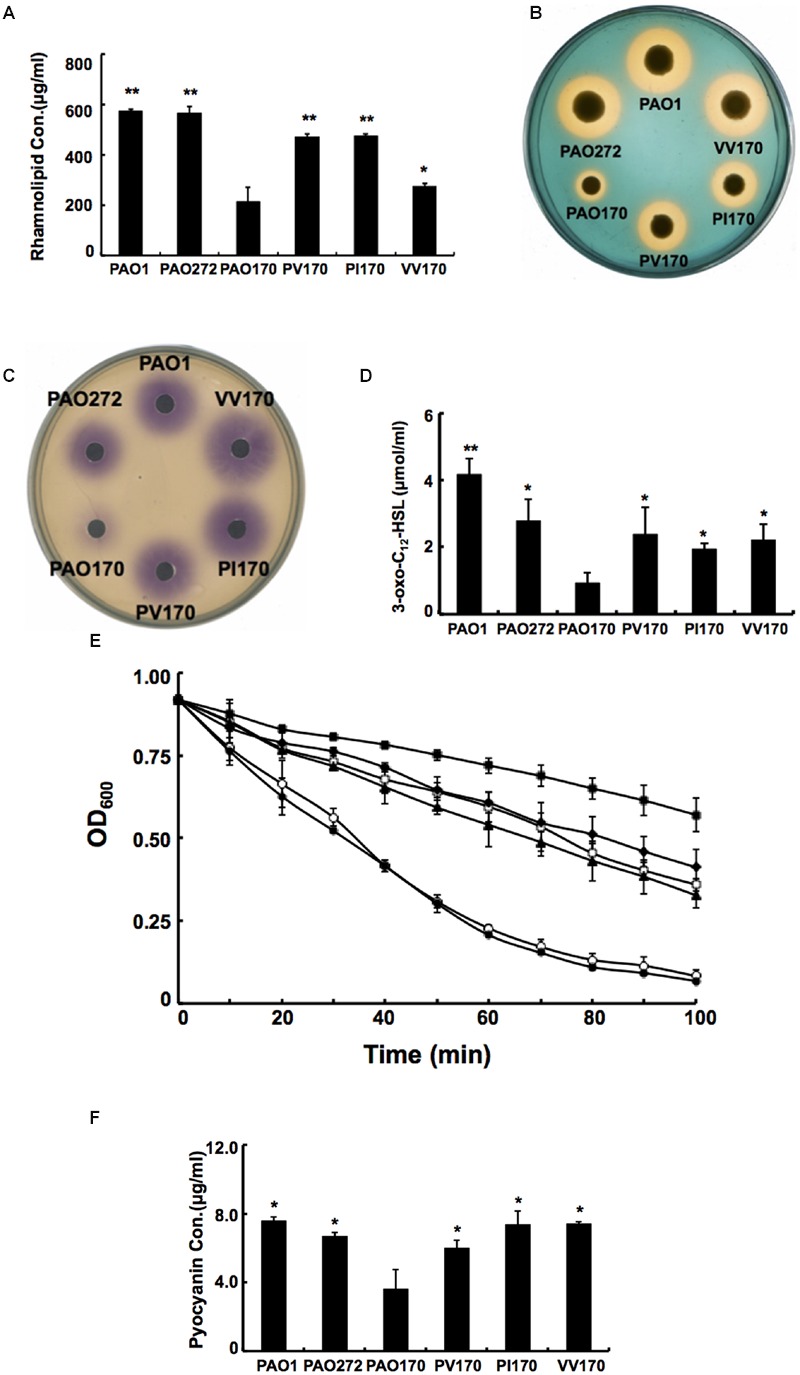
**Characterization of several exo-products produced by *P. aeruginosa* strains. (A)** Secretion of rhamnolipids by *P. aeruginosa* strains. **(B)** Siderophore secretion by *P. aeruginosa* strains. **(C)** The C_4_-HSL produced by *P. aeruginosa* strains. **(D)** The 3-oxo-C_12_-HSL produced by *P. aeruginosa* strains. **(E)** LasA protease activities in culture supernatants of *P. aeruginosa* strains. Filled circle indicates strain PAO1; empty diamond indicates strain PAO272; filled triangle indicates strain PV170; empty square indicates strain VV170; filled diamond indicates strain PI170 and filled square indicates strain PAO170. **(F)** Pyocyanin in culture supernatants of *P. aeruginosa* strains. Data are the mean ± standard deviation of triplicate measurements. Pair-wise comparisons were made between mutant strain PAO170 and wild-type strain PAO1 or complemented strains, such as, PV170, PI170 and VV170, by Student’s *t*-test. ^∗∗^, Highly significant difference, *P* < 0.01. ^∗^, Significant difference, *P* < 0.05.

Given that deletion of *fabV* decreased the activity of the ENR of *P. aeruginosa* significantly, decreased production of several exo-products, which are synthesized using the intermediates in the fatty acid synthetic pathway, would be expected in PAO170 mutant strains. We first looked at levels of rhamnolipids produced by *P. aeruginosa* strains. Rhamnolipids are secreted surfactant glycolipids assembled by rhamnosyltransferase using L-rhamnose and 3-hydroxydecanoyl-ACP from the FAS pathway (Deziel et al., 2003; Zhu and Rock, 2008). Rhamnolipid quantification by colorimetric detection of rhamnose showed a twofold decrease in the *fabV* mutant PAO170, while the amount of rhamnolipid produced by complemented strain PAO170/pSRK-PV was up to 80% of that of the wild-type strain PAO1 (**Figure 5A**).

Pyoverdine is the dominant siderophore of *P. aeruginosa* and is assembled from tetradecanoyl-ACP (Drake and Gulick, 2011; Hannauer et al., 2012). The siderophore produced by *P. aeruginosa* strains was examined on LB-CAS indicator plates. Yellow-orange halos around the cultures indicate where the siderophores have sequestered Fe^3+^ away from the blue CAS-Fe^3+^ complex. The halo produced by the PAO170 mutant strain was much smaller than that of the wild-type strain: its average diameter was only a quarter of that of the wild-type strain. After complementation with plasmid pSRK-PV, the average diameter of the yellow-orange halo of the PAO170 mutant strain increased to one half that of the wild-type strain (**Figure 5B**).

The *rhl* and *las* QS signal molecules, N-butanoyl-L-homoserine lactone (C_4_-HSL) and N-(3-oxododecanoyl)-L-homoserine lactone (3-oxo-C_12_-HSL), are synthesized from S-adenosylmethionine and butanoyl-ACP or 3-oxododecanoyl-ACP, respectively (Parsek et al., 1999; Hoang et al., 2002). Both acyl-ACPs are intermediates in the FAS pathway. Therefore, we also measured the levels of both QS signal molecules produced by *P. aeruginosa* strains. The *rhl* and *las* QS signals were extracted from culture supernatants of *P. aeruginosa* strains. The acyl-HSLs were detected using an agar overlay of a *C. violaceum* reporter strain CV026, which produces a purple halo in response to acyl-HSLs. The purple halo around PAO170 mutant strain was weak and small in comparison to that of the wild-type strain (**Figure 5C**). Production of acyl-HSLs was increased to wild-type levels by complementation with strain PAO170/pSRK-PV. The 3-oxo-acyl-HSL was detected using HPLC. The level of 3-oxo-acyl-HSL showed a threefold decrease in the *fabV* mutant in comparison to that of the wild-type strain and there was highly significant difference between strain PV170 and PAO170 (*P* < 0.01). Complemented strain PAO170/pSRK-PV produced more 3-oxo-acyl-HSL than the *fabV* mutant, but not to the level of the wild-type strain (**Figure 5D**).

The PQS signal molecules also utilize 3-ketoacyl medium-chain fatty acid metabolites to synthesize 2-heptyl-3-hydroxy-4-quinolone (PQS; Girard and Bloemberg, 2008; Williams and Camara, 2009). We thus analyzed the levels of PQS in supernatant extract in *P. aeruginosa* strains. The level of PQS was reduced to a trace in the supernatant extract when *fabV* was deleted. In contrast, PQS production was markedly enhanced in the complemented strain (data not shown).

We also complemented the PAO170 mutant with other ENR encoding genes, such as *P. aeruginosa fabI* or *V. cholerae fabV*, and all complemented strains produced more rhamnolipids, pyoverdine, AHLs and PQS than the *fabV* mutant (**Figures 5A–D**).

### Addition of Exogenous AHLs Restored the Ability of the *fabV* Strain to Produce Virulence Factors

The QS systems, including *rhl, las* and PQS, regulate the ability of *P. aeruginosa* to produce virulence factors (e.g., LasA/LasB and alkaline proteases, phospholipases, lipases, exotoxin A, rhamnolipid, pyocyanin, and others; Girard and Bloemberg, 2008; Williams and Camara, 2009). Thus, reduction of QS signal molecules should decrease production of these virulence factors. To confirm this, we tested the production of LasA and pyocyanin by *P. aeruginosa* strains. The data showed that the activity of LasA in PAO170 mutant was much lower than that in the wild-type strain, and the amount of pyocyanin produced by PAO170 strain was about 50% of that produced by the wild-type strain (**Figures 5E,F**). However, complementation of PAO170 mutant strain with ENR genes increased the activity of LasA and the amount of pyocyanin (**Figures 5E,F**).

**FIGURE 6:**
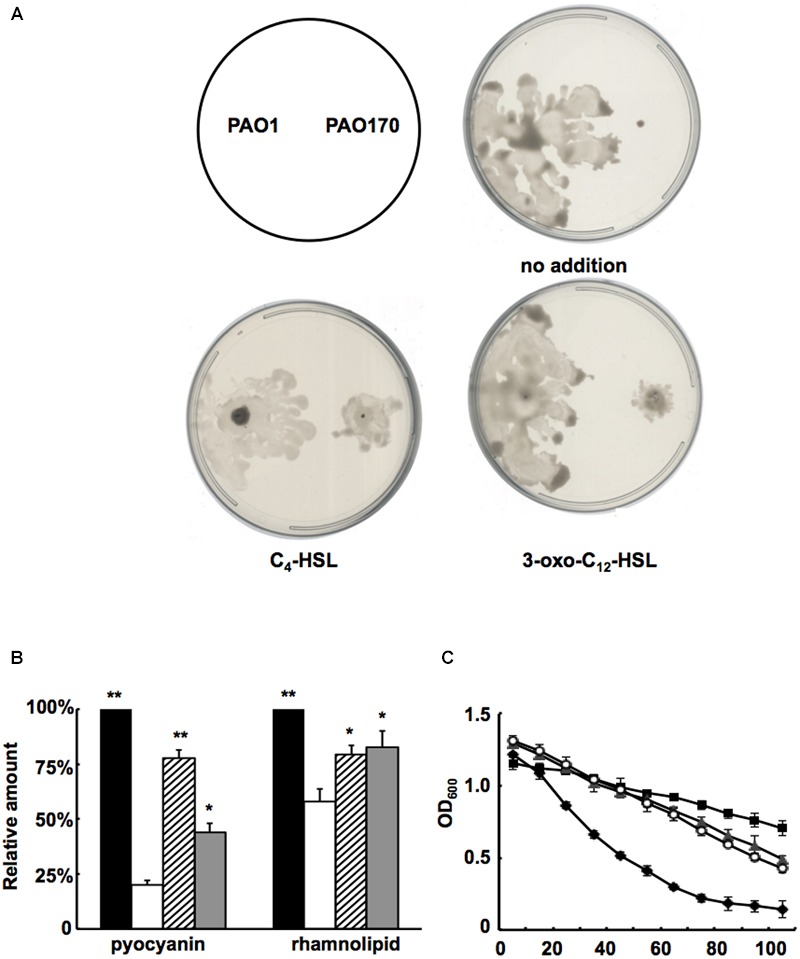
**Effect of exogenous AHLs on swarming motility and production of several virulence factors by the *P. aeruginosa fabV* deletion strain. (A)** Effect of exogenous AHLs on swarming motility of the *P. aeruginosa fabV* deletion strain. **(B)** Effect of exogenous AHLs on pyocyanin and rhamnolipid secretion by the *P. aeruginosa fabV* deletion strain. Black bar indicates the culture of strain PAO1; white bar indicates the culture of strain PAO170; hatched bar indicates the culture of strain PAO170 after addition of C_4_-HSL; gray bar indicates the culture of strain PA0170 after addition of 3-oxo-C_12_-HSL. Data are the mean ± standard deviation of triplicate measurements. Pair-wise comparisons were made between mutant strain PAO170 and wild-type strain PAO1 by Student’s *t*-test. ^∗∗^, Highly significant difference, *P* < 0.01. ^∗^, Significant difference, *P* < 0.05. **(C)** LasA protease activities in culture supernatants of *P. aeruginosa* strains after addition of exogenous AHLs. Filled diamond indicates the culture of strain PAO1; empty circle indicates the culture of strain PA170 after addition of C_4_-HSL; filled triangle indicates the culture of strain PV170 after addition of 3-oxo-C_12_-HSL; filled square indicates the culture of strain PA170.

Based on the above study, it would be expected that, on addition of QS signal molecules to cultures of *P. aeruginosa* strains, production of some virulence factors that are regulated by the QS system would be restored. First, we added C_4_-HSL or 3-oxo-C_12_-HSL to swarming plates and tested the swarming motility of the *fabV* mutant strain. Exogenous addition of both QS signals to the swarming plate restored the ability of the *fabV* mutant to form a typical swarming pattern after 24 h of incubation, although the pattern was still smaller than that of the wild type (**Figure 6A**). We also examined production of pyocyanin, rhamnolipid and LasA protease by PAO170 mutant strain after exogenous addition of QS signals. The data showed that, although not up to the levels of the wild-type strain, the production of all these virulence factors was increased to a certain extent (**Figures 6B,C**). These findings indicated that deletion of *fabV* reduced the activity of ENR in *P. aeruginosa*, subsequently depressed the production of QS signals and finally led to reduced virulence factors in *P. aeruginosa*.

## Discussion

Complementation with any one of the known triclosan-resistant ENR encoding genes, such as *V. cholerae fabV, E. faecalis fabK, B. subtilis fabL* or *P. aeruginosa fabV*, restored triclosan resistance in the *fabV* deleted strain to the level of the wild-type strain, which confirms that FabV is responsible for the inherent triclosan resistance of *P. aeruginosa*. This confirms that a triclosan-resistant ENR may confer high resistance to triclosan on a bacterium. *E. faecalis* has a triclosan-resistant ENR encoding gene, *fabK*, and is resistant to triclosan (the MIC of triclosan is 10 μg/ml), but it has been demonstrated that the *E. faecalis* FabK does play a role in the inherent triclosan resistance of this bacterium (Zhu et al., 2013). Moreover, *S. pneumoniae, B. subtilis* and *V. cholerae* also possesses triclosan-resistant ENR encoding genes (*fabK, fabL* and *fabV*; Heath et al., 2000; Marrakchi et al., 2003; Massengo-Tiasse and Cronan, 2008), and expression of *S. pneumoniae fabK, B. subtilis fabL* or *V. cholerae fabV* greatly increased the resistance of *E. coli* to triclosan, but the MIC of triclosan for *S. pneumoniae, B. subtilis* and *V. cholerae* is only about 2–4 μg/ml, which is much lower than that of *P. aeruginosa.* This suggests that triclosan may target a cellular process unrelated to fatty acid synthesis in these bacteria.

The ENRs reduce *trans*-2-enoyl-ACPs to the fully saturated ACP species in the last step of the elongation cycle in the synthesis of bacterial FAs (White et al., 2004). In *E. coli*, there is a single, NADH-dependent ENR isoform, FabI, which reduces all of the enoyl intermediates of the pathway and is essential to cell growth and survival. Physiological studies have shown that *E. coli* FabI plays a determining role in completing rounds of fatty acid elongation and is feedback inhibited by long-chain ACPs (Heath and Rock, 1995). Unlike *E. coli, B. subtilis, E. faecalis* and *P. aeruginosa* have two ENRs (Heath et al., 2000; Zhu et al., 2010, 2013), which can be either of the same or of different protein families. However, although both ENR isozymes in these bacteria catalyze reduction of *trans*-2-enoyl-ACPs to the saturated ACP species, the physiological rationale for this duplication of enzyme activity has been poorly explored. Recently, the functions of both FabI and FabK in *E. faecalis* were characterized and the results showed that each enzyme has a discrete physiological role. FabI is essential for *E. faecalis* growth and plays the key role in the fatty acid synthetic pathway, whereas deletion of FabK does not affect growth of *E. faecalis* and FabK only modulates the composition of phospholipid acyl chains (Zhu et al., 2013). *P. aeruginosa* possesses FabI and FabV, two ENR isozymes. Although neither the *fabI* nor the *fabV* gene is essential to *P. aeruginosa*, the deletion of *fabV* produced more 3-hydroxy fatty acids and fewer fatty acids, and this strain grew much more slowly than the wild-type strain. Moreover, *fabV* deletion attenuated swarming motility, decreased production of rhamnolipid, pyoverdine and AHLs, and reduced virulence factors. All these results suggest that FabV is the main ENR of *P. aeruginosa*. However, mutation of *fabI* did not affect *P. aeruginosa* growth or the fatty acid profiles. This indicates that, unlike FabK in *E. faecalis*, FabI does not play a role in modulating fatty acid composition at least under the growth conditions we have tested. However, the *fabI* mutant showed reduced ENR activity in cell extracts and produced fewer AHLs (Hoang and Schweizer, 1999; Zhu et al., 2010). Thus, we speculate that *fabI* may be required to maintain sufficient ENR activity in *P. aeruginosa*.

*Pseudomonas aeruginosa* is a gram-negative bacterium and produces lipid A in its outer membrane (Pier, 2007). When we determined the total cell fatty acid composition in *P. aeruginosa* strains by GC-MS, we found that deletion of *fabV* led *P. aeruginosa* to produce more 3-hydroxydecanoic and 3-hydroxydodecanoic acids, which are the main components of lipid A of *P. aeruginosa*. This deletion of *fabV* changed the production of lipid A in *P. aeruginosa*. However, we also demonstrated that increasing 3-hydroxy fatty acids was not the main reason for the weak growth of *P. aeruginosa*. In the bacterial fatty acid synthetic pathway, the ENRs are responsible for pulling *trans*-2-enoyl-ACPs to the fully saturated ACP species. However, the conversion of 3-hydroxyacyl-ACPs to *trans*-2-enoyl-ACPs is catalyzed by 3-hydroxyacyl-ACP dehydrase (FabA and FabZ) and is an equilibrium reaction (Heath and Rock, 1995). Thus, larger amounts of 3-hydroxy fatty acids may accumulate in a cell when the level of *P. aeruginosa* ENRs is decreased.

The three dominant QS signal molecules from the *las, rhl*, and PQS systems, all of which contain fatty acid moieties, together form a complex, cell density-dependent regulatory circuit, which not only regulates the production of many exo-products in *P. aeruginosa*, but also is involved in bacterial motility (Girard and Bloemberg, 2008; Williams and Camara, 2009). The deletion of *fabV* led *P. aeruginosa* to reduce the production of QS signal molecules and many exo-products, and caused *P. aeruginosa* to fail to swarm on semisolid plates. However, exogenous supplementation of AHLs, C_4_-HSL or 3-oxo-C_12_-HSL increased the production of pyocyanin, rhamnolipid and LasA protease in the *fabV* mutant, and restored *fabV* mutant swarming on semisolid plates. These findings indicated that deletion of *fabV* caused decreased production of AHL signals, attenuated QS systems, and subsequently reduced the virulence factors of *P. aeruginosa*.

Deletion of *fabV* attenuated not only growth but also QS and many virulence factors. Therefore, inhibition of FabV in *P. aeruginosa* is a compelling strategy for use in the development of new antimicrobial agents.

## Author Contributions

Y-HH cloned ENR genes, constructed several ENRs expression vectors, did complementation experiments with ENRs expression vectors, and carried out biochemical studies. J-SL constructed fabV mutants and tested the growth of mutants in LB medium. J-CM carried out experiments on the pathogenesis of *P. aeruginosa*. H-HW conceived of the study, and participated in its design and coordination and helped to draft the manuscript. All authors read and approved the final manuscript.

## Conflict of Interest Statement

The authors declare that the research was conducted in the absence of any commercial or financial relationships that could be construed as a potential conflict of interest.
