# Supplementary material for: Functional Characterization of Triclosan-Resistant Enoyl-acyl-carrier Protein Reductase (FabV) in Pseudomonas aeruginosa
Source: Front Microbiol. 2016 Nov 29;7:1903. doi: 10.3389/fmicb.2016.01903 (PMC5126088; doi:10.3389/fmicb.2016.01903)
Supplement: Supplementary file 1 [file Data_Sheet_1.DOCX]

**Functional characterization of triclosan-resistant enoyl-acyl-carrier protein reductase (FabV) in *Pseudomonas aeruginosa***

Yong-Heng Huang^1^, Jin-Shui Lin^2^, Jin-Cheng Ma^1^, Hai-Hong Wang^1^*

Supplementary Files

**Table S1. Bacterial strains and plasmids used in this study**

| Bacterial strains | Relevant characteristics*^a^* | Source |
| --- | --- | --- |
| *E. coli* |  |  |
| DH-5α | F^-^ *deoR* *endA1 gyrA96 hsdR17*(r_K_^-^m_K_^+^) *recA1* *relA1* *supE*44 *thi-1* Δ(*lacZYA-argF*)*U*169(φ80*lacZ*ΔM15) | Lab stock |
| *P. aeruginosa* | |  |
| PAO1 | Wild-type strain | ATCC |
| PAO170 | PAO1 *fabV*::Gm^r^ | (Zhu et al., 2010) |
| PAO272 | PAO1 *fabI*::Gm^r^ | (Zhu et al., 2010) |
| PI170 | PAO170/ pSRK-PI | This study |
| PV170 | PAO170/ pSRK-PV | This study |
| VV170 | PAO170/ pSRK-VV | This study |
| EK170 | PAO170/ pSRK-EK | This study |
| BL170 | PAO170/ pSRK-BL | This study |
| Plasmids |  |  |
| pMD19 | Amp^r^, TA cloning vector | Takara |
| pYH1 | Amp^r^, *P. aeruginosa* *fabI* gene was cloned into plasmid pMD19 | This study |
| pYH2 | Amp^r^, *P. aeruginosa* *fabV* gene was cloned into plasmid pMD19 | This study |
| pYH3 | Amp^r^, *V. cholerae fabV* gene was cloned into plasmid pMD19 | This study |
| pYH4 | Amp^r^, *E. faecalis* *fabK* gene was cloned into plasmid pMD19 | This study |
| pYH5 | Amp^r^, *B. subtilis fabL* gene was cloned into plasmid pMD19 | This study |
| pSRK-Km | Km^r^, broad-host-range expression vector containing *lac* promoter and *lacI*^q^, *lacZ*α^+^ | (Khan et al., 2008) |
| pSRK-PI | Km^r^, *P. aeruginosa* *fabI* gene was cloned into plasmid pSRK-Km | This study |
| pSRK-PV | Km^r^, *P. aeruginosa* *fabV* gene was cloned into plasmid pSRK-Km | This study |
| pSRK-VV | Km^r^, *V. cholerae fabV* gene was cloned into plasmid pSRK-Km | This study |
| pSRK-EK | Km^r^, *E. faecalis* *fabK* gene was cloned into plasmid pSRK-Km | This study |
| pSRK-BL | Km^r^, *B. subtilis fabL* gene was cloned into plasmid pSRK-Km | This study |

**Table S2. Primers for ENR genes cloning**

| Name | Sequences (5'→3') |
| --- | --- |
| *PafabV-*Nde I | ACCGAGGTTcatatgATCATCAAACCGCGC |
| *pafabV-*Hind III | AGGCaagcttGTCGCTGAAAACGCGAAC |
| *pafabI -*Nde I | CGGcatatgGGATTTCTCACAGGAA |
| *pafabI-* Hind III | GGGaagcttAGTCGTCGTCCAGCGG |
| *vcfabV-*Nde I | AAGGAGGAGACcatatgATC |
| *vcfabV-*Hind III | CTGCaagcttACTCGATATC |
| *bsfabL-* Nde I | GGAGATGAGGAcatatgGAAC |
| *bsfabL-*BamH I | TTACGggatccCTTAAACGAGCAGTGAG |
| *enfabK-*Nde I | GGGAATTCcatatgAAGTGTACTTATCTTAGAAC |
| *enfabK-*Hind III | GACTGGaagcttAGCCCCAACGCTGATTCATC |

Note: lower case letters show the restriction sites.

**Figure S1**

**Fig. 1S. Growth inhibition of *P. aeruginosa* strains by triclosan.** The concentration of triclosan is shown below each plate. PAO1 indicates *P. aeruginosa* wild-type strain PAO1; PAO170 indicates *P.aeruginosa fabV* deletion strain; PAO272 indicates *P. aeruginosa fabI* deletion strain; BL170 indicates strain PAO170 carrying plasmid pSRK-BL; EK170 indicates strain PAO170 carrying plasmid pSRK-EK.

**Figure S2**

**Fig. S2. Swarming motility of *P. aeruginosa* strains.** Swarming motility patterns of *P.aeruginosa* strains after incubation at 30 °C for 24–72 h. PAO1 indicates *P. aeruginosa* wild-type strain PAO1; PAO170 indicates *P. aeruginosa fabV* deletion strain; BL170 indicates strain PAO170 carrying plasmid pSRK-BL; EK170 indicates strain PAO170 carrying plasmid pSRK-EK.
